# Ogilvie Syndrome: A Therapeutic Challenge

**DOI:** 10.7759/cureus.103754

**Published:** 2026-02-17

**Authors:** Ana Sofia Reis, Ana Rita Antunes, Lígia R Santos

**Affiliations:** 1 Internal Medicine, Unidade Local de Saúde de Gaia e Espinho, Vila Nova de Gaia, PRT

**Keywords:** elderly, intestinal motility disorders, intestinal pseudo-obstruction, neostigmine, ogilvie syndrome

## Abstract

Ogilvie syndrome, or acute colonic pseudo-obstruction, is a rare disorder characterized by colonic dilation without mechanical obstruction. It primarily affects elderly or hospitalized patients and can lead to serious complications, including ischemia and perforation. Management typically begins with conservative measures, but refractory cases may require endoscopic or pharmacologic interventions. Current literature lacks robust evidence to guide the optimal therapeutic approach. We present a case that demonstrates practical management strategies, mitigates therapeutic inertia, and supports continuous medical education.

## Introduction

Ogilvie syndrome is a functional colonic distension whose pathophysiology remains unclear, but current evidence suggests an alteration in the autonomic nervous system resulting in colonic atony and pseudo-obstruction. It is more prevalent among hospitalized patients, particularly the elderly, with multiple comorbidities, electrolyte imbalances, or recent surgical procedures [1]. The most severe complications include intestinal ischemia and perforation [2]. Treatment is primarily conservative, consisting of correction of electrolyte abnormalities, bowel rest, and nasogastric and rectal tube decompression. In cases refractory to conservative measures, additional interventions may be required, including endoscopic decompression (colonoscopy), often used to reduce cecal diameter and prevent perforation, and pharmacologic treatment with intravenous neostigmine (a parasympathomimetic agent) that effectively resolves colonic dilation. If these fail or signs of perforation/ischemia occur, surgical intervention (e.g., cecostomy, resection) is required [2,3]. While uncommon, Ogilvie syndrome carries a high risk of severe complications and significant morbidity. Interventional treatments are associated with significantly higher complication rates, reaching up to approximately 60%. Overall, medical complications after any treatment occur in 45% of cases, while procedure-related complications (colonoscopy or surgery) occur in 16%. The overall treatment-associated mortality is 7.7%, increasing with procedural invasiveness. In cases requiring surgery after failure of conservative management, mortality may reach 60% when perforation or colonic ischemia is present [4]. Early recognition and optimization of medical management, including correction of precipitating factors, are the cornerstone of treatment. The main therapeutic challenge arises in cases unresponsive to initial measures, as illustrated by the case presented here.

## Case presentation

**Figure 1:**
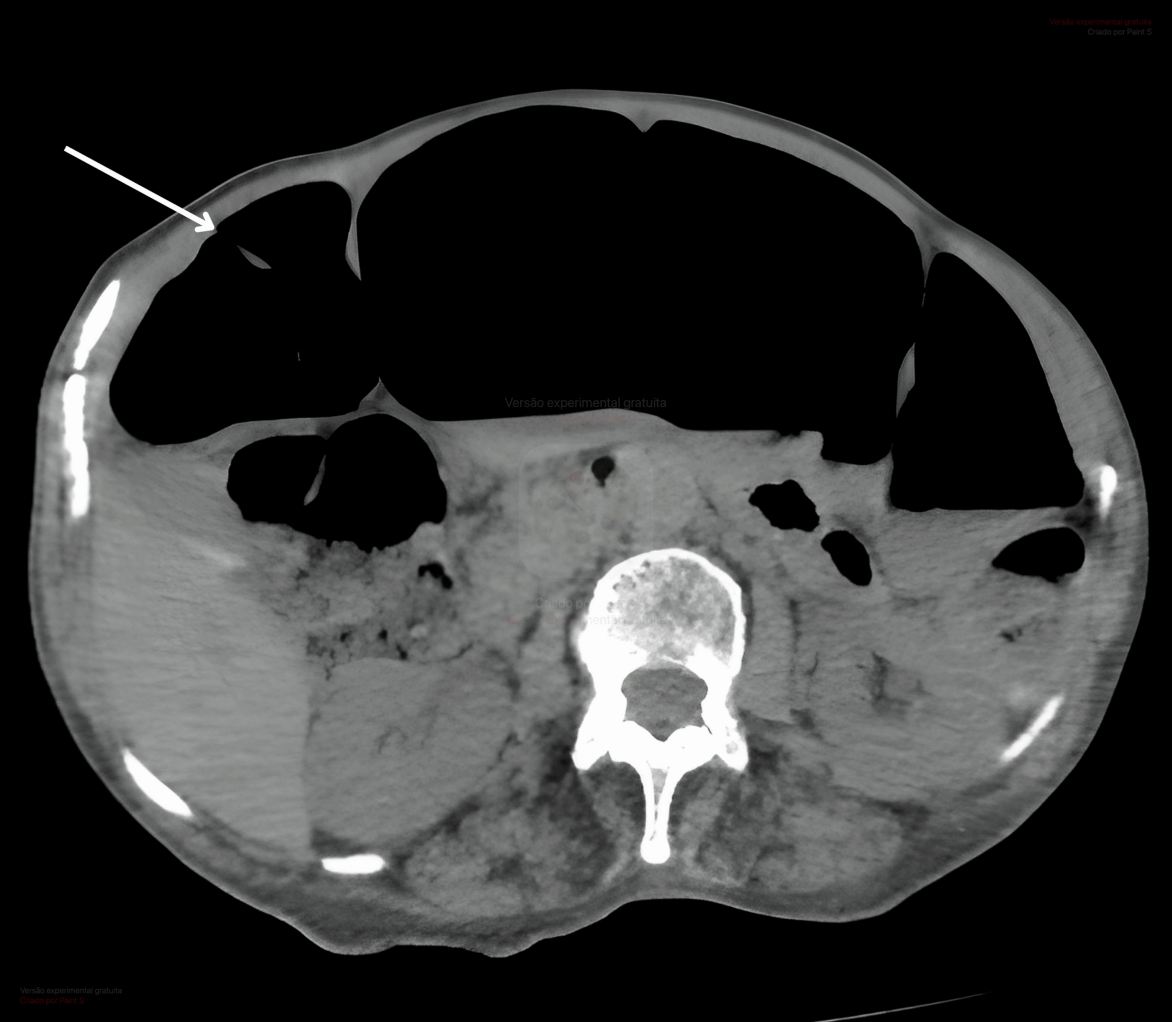
Abdominal CT showing colonic dilatation, predominantly involving the cecum and ascending colon, consistent with acute colonic pseudo-obstruction.

**Figure 2:**
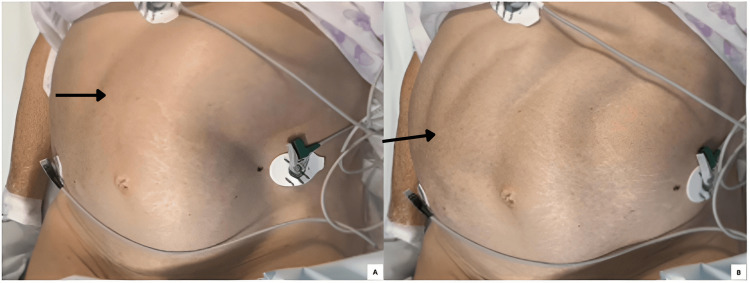
(A, B) Motility of the intestinal loops (arrows) during neostigmine infusion.

A 70-year-old woman with no significant medical history, recent hospitalization, surgery, immobility, or acute illness presented to the emergency department with several weeks of constipation, abdominal pain, and postprandial vomiting. Physical examination revealed a distended abdomen, tympanic to percussion, with mild tenderness on palpation. Laboratory tests showed hypokalemia (2.9 mmol/L; reference range: 3.5-5 mmol/L). Thyroid dysfunction and *Clostridioides difficile* infection were excluded. The patient had no history of systemic disease or neurological disorders and was not taking any medications known to precipitate these symptoms, such as opioids, anticholinergics, or calcium channel blockers. Abdominal CT demonstrated marked colonic distension (Figure 1), with the cecum measuring 11 cm, and associated small bowel distension, but no evidence of mechanical obstruction. The patient was admitted and started on medical management, including potassium correction, bowel rest, and nasogastric decompression. However, the condition proved refractory to this approach. Colonoscopic decompression was subsequently performed with initial improvement, but symptoms recurred shortly thereafter. Ultimately, intravenous neostigmine was administered, resulting in complete resolution of symptoms. Motility of intestinal loops during neostigmine infusion can be seen in Figure 2. The patient was discharged with restored bowel function and was tolerating oral intake. Treatment with prokinetics and laxatives was maintained for the prevention of relapses.

## Discussion

Acute colonic pseudo-obstruction represents a significant clinical challenge due to its unpredictable course and potential for life-threatening complications. The condition often arises from a disruption in the normal neural control of the large intestine, leading to impaired motility and progressive bowel distension [4]. Clinical manifestations typically include progressive abdominal enlargement, discomfort, nausea, and difficulty with stool passage. Laboratory evaluation may reveal electrolyte disturbances that both predispose and exacerbate impaired gastrointestinal function. Imaging studies are essential for diagnosis, as they allow assessment of colonic dimensions and exclude both obstructive lesions and local complications, such as perforation. Notably, enlargement of the cecum beyond critical thresholds leads to vascular compromise and perforation, emphasizing the importance of timely intervention, as the onset of complications requiring surgical intervention significantly increases mortality rates. In uncomplicated cases, mortality is lower, though morbidity remains substantial [5].

Initial management prioritizes supportive measures and correction of reversible factors, supporting the hypothesis that acute colonic pseudo-obstruction may be associated with impaired parasympathetic activity. The main challenge, aside from cases with complications requiring surgery (peritonitis, intestinal ischemia or perforation, hemodynamic instability associated with disease progression, and cecal diameter greater than 12 cm), arises when initial conservative management fails. In cases refractory to treatment with conservative measures, pharmacologic agents that enhance parasympathetic activity may provide rapid and sustained improvement. Careful monitoring during administration is crucial due to the possibility of cardiovascular or respiratory adverse events. Our case is consistent with the conclusions reported by Turégano-Fuentes et al. [6], in which neostigmine therapy was shown to be effective, supporting the assumption that acute colonic pseudo-obstruction (Ogilvie syndrome) is associated with excessive suppression of parasympathetic activity.

## Conclusions

It is widely accepted that a stepwise approach to care should be adopted. Conservative management aimed at correcting potentially reversible disturbances should always be attempted as the first-line strategy. It is also well recognized that surgical intervention is reserved for cases presenting with severe complications. The challenge arises when conservative measures fail in the absence of complications. The literature is not unanimous on the optimal management in such scenarios; however, the most widely accepted approaches include endoscopic therapy and treatment with parasympathomimetic agents. Based on the experience from the case presented, pharmacological therapy appears to be an effective option in selected patients. Detailed case reports such as the present one provide valuable clinical insight, contributing to the accumulation of practical knowledge, informing decision-making, and potentially enhancing the standard of care in similar clinical scenarios.
